# 1016. Real-World Use of Omadacycline in Physician Office Infusion Centers (POICs)

**DOI:** 10.1093/ofid/ofac492.857

**Published:** 2022-12-15

**Authors:** Lucinda J Van Anglen, Claudia P Schroeder, Kimberly A Couch

**Affiliations:** Healix Infusion Therapy, LLC, Sugar Land, Texas; Healix Infusion Therapy, LLC, Sugar Land, Texas; Healix Infusion Therapy, LLC, Sugar Land, Texas

## Abstract

**Background:**

Omadacycline (OMC) is approved for the treatment of community-acquired bacterial pneumonia and acute bacterial skin and skin structure infections. Real-world data on the use of OMC are limited. We present a multicenter observational review of OMC outpatient use in Infectious Disease POICs.

**Methods:**

Medical records of patients (pts) receiving intravenous OMC from May 2019 to April 2022 from 5 POICs were included in this ongoing study. Data included demographics, diagnosis, medical history, microbiology, OMC regimen, adverse events (AEs), health care utilization and clinical outcomes. Clinical success was defined as complete or partial symptom resolution at completion of OMC with oral antibiotics as needed. Persistent or recurrent infection and premature discontinuation of OMC were deemed non-success. Indeterminate outcomes were excluded in outcome assessment. Chi Square, Fisher’s exact, and t-test were used to identify characteristics associated with clinical outcome.

**Results:**

Overall, 37 pts (mean age: 61±13 yrs, 65% male) were identified. Infections treated were 62% bone and joint (BJI), 24% complicated skin and skin structure infections (CSSSI), 11% pulmonary nontuberculous mycobacterial (NTM) and 3% diverticulitis. A total of 61 pathogens were identified in 30 pts of which 83% had ≥1 Gram-positive isolate. Polymicrobial pathogens were reported in 13/30 pts (43%). Ten pts (27%) received concomitant IV antibiotics. Median duration of OMC therapy was 38 days (IQR, 21-47). OMC was initiated in the POIC without prior hospitalization in 62% of pts. Overall clinical success was 75% (24/31). Non-success due to persistent infection was reported in 5 pts (3 BJI, 2 CSSSI), of which 4 were hospitalized. Three additional pts were deemed non-successful due to early discontinuations (2 difficulty with administration, 1 AE) (Fig 1). Six pts were non-evaluable for outcome. Ten pts reported 14 AEs, most commonly nausea (4 pts) and elevated liver function tests (3 pts).

**Figure 1.**
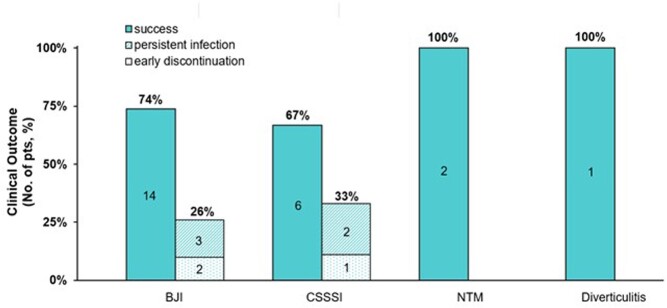
Clinical outcome of omadacycline by infection type

**Conclusion:**

These real-world results support the outpatient use of omadacycline in additional, difficult to treat, complicated infections, including bone and joint infections.

**Disclosures:**

**Lucinda J. Van Anglen, PharmD**, Merck & Co.: Grant/Research Support|Paratek: Grant/Research Support.

